# Autoregulatory Feedback Controls Sequential Action of *cis*-Regulatory Modules at the *brinker* Locus

**DOI:** 10.1016/j.devcel.2013.08.010

**Published:** 2013-09-16

**Authors:** Leslie Dunipace, Abbie Saunders, Hilary L. Ashe, Angelike Stathopoulos

**Affiliations:** 1 Division of Biology, California Institute of Technology, Pasadena, CA 91125, USA; 2 Faculty of Life Sciences, University of Manchester, Manchester M13 9PT, UK

## Abstract

*cis*-regulatory modules (CRMs) act sequentially to regulate temporal expression of genes, but how the switch from one to the next is accomplished is not well understood. To provide insight, here we investigate the *cis*-regulatory system controlling *brinker* (*brk*) expression in the *Drosophila* embryo. Two distally located CRMs support expression at different times, while a promoter-proximal element (PPE) is required to support their action. In the absence of Brk protein itself or upon mutagenesis of Brk binding sites within the PPE, the late-acting CRM, specifically, is delayed. This block to late-acting CRM function appears to be removed when the early-acting CRM is also deleted. These results demonstrate that autoregulatory feedback is necessary for the early-acting CRM to disengage from the promoter so that the late-acting CRM may act. Autoregulation may be a commonly used mechanism to control sequential CRM action necessary for dynamic gene expression throughout the course of development.

## Highlights

• Two CRMs support spatiotemporally distinct expression of *brinker* in early embryo
• A promoter-proximal element (PPE) is required to support action of both CRMs
• Brk protein acts at the PPE to facilitate the switch from early- to late-acting CRM
• Autoregulation may be a common mechanism used to control sequential CRM action

## Teaser

*cis*-regulatory modules within a given gene that drive overlapping patterns of expression are frequently called redundant “shadow” enhancers. Dunipace et al. find that, in fact, some such enhancers act sequentially to refine expression patterns. A promoter-proximal element uses transcription factor autoregulation to coordinate the handoff from one enhancer to another.

## Introduction

Many genes are pervasively expressed throughout development by the sequential action of *cis*-regulatory modules (CRMs). Studies of sequentially acting CRMs have been most clearly characterized through deletions made in the context of large reporter transgenes, encompassing the majority, if not the entirety, of a gene’s *cis*-regulatory information (Lee et al., 2007; Pfeffer et al., 2002). However, little is known regarding how the handoff from one CRM to the next is accomplished or whether this process is regulated. To provide insight, here we have investigated the *cis*-regulatory system controlling dynamic embryonic expression of the gene *brinker* (*brk*) in *Drosophila melanogaster*.

The *brk* gene is continuously expressed during development, and its product plays an important role in cell patterning (Jaźwińska et al., 1999a). *brk* encodes a transcriptional repressor and acts, at least in part, to refine gene expression downstream of BMP signaling. Several previous studies have focused on the identification and initial characterization of CRMs that act to control *brk* gene expression (Müller et al., 2003; Yao et al., 2008). Five distinct CRMs were identified upstream of the *brk* gene that support expression in the wing disc (Yao et al., 2008). It was suggested that this set of CRMs works coordinately to control *brk* expression, in that their combined output is thought to support the *brk* pattern in the wing disc. Conversely, just two CRMs have been identified that support *brk* early embryonic expression along the dorsal-ventral axis: one present ∼10 kb upstream of the gene and the other present ∼8 kb downstream (Hong et al., 2008; Markstein et al., 2004; Ozdemir et al., 2011). These CRMs acting in the embryo were also found to support similar expression within lateral stripes along the dorsal-ventral axis of the embryo. Based on their similarity of expression, it was suggested that they provide evolutionary robustness (Hong et al., 2008). However, no previous study has examined the function of individual *brk* gene-associated CRMs in the context of the gene locus or examined their temporal expression profiles.

In this study, we have focused on dissecting the role of individual CRMs associated with the *brk* locus in the early embryo with the goal of providing understanding of the regulation of gene expression in general. Our results demonstrate that the embryonic CRMs acting at the *brk* locus support temporally distinct patterns. In addition, our data show that autoregulatory feedback is the mechanism used in the early embryo to switch from the early-acting CRM to the late-acting CRM at this locus. Specifically, we found that Brk binding to the promoter-proximal sequence is important for managing this exchange. These results suggest that autoregulation may be a commonly used mechanism to support dynamic and continuous gene expression by controlling the timing of association of sequentially acting CRMs with the promoter.

## Results and Discussion

Our previous ChIP-seq studies examined transcription factor occupancy in the genome, identifying three regions of occupancy for the bHLH transcription factor Twist at the *brk* locus: one region located promoter proximally and two regions located at a distance (Figure S1A available online) (Ozdemir et al., 2011). The region near the promoter, the promoter-proximal element (PPE), failed to support gene expression by standard reporter gene assay (Figures S1E–S1E″). In contrast, the distally located regions (i.e., 5′ and 3′ CRMs) have been shown to support gene expression within lateral stripes along the dorsal-ventral axis (Hong et al., 2008; Markstein et al., 2004; Ozdemir et al., 2011). Previous studies have highlighted the similarity of the patterns supported by these CRMs (Hong et al., 2008).

When expression supported by the 5′ and 3′ CRMs was examined with temporal resolution at three specific time points, (1) precellularization, (2) cellularization, or (3) gastrulation, it became clear that these CRMs support different expression profiles. Similar reporter expression is supported by each CRM in the early embryo precellularization (i.e., thin ventrolateral stripe; Figures S1C and S1D). However, at cellularization, the 3′ CRM supports a broad lateral pattern, whereas the 5′ CRM pattern remains thin, limited to ventrolateral regions (Figures S1D′ and S1C′, respectively). At gastrulation, the patterns supported also differ: the 3′ CRM supports broad ectodermal expression throughout the trunk, whereas the 5′ CRM supports only minimal expression at the anterior and posterior ends (Figures S1D″ and S1C″, respectively). Therefore, standard reporter assays suggest these two CRMs drive similar expression in the early embryo precellularization but different expression at later stages.

**Figure 1:**
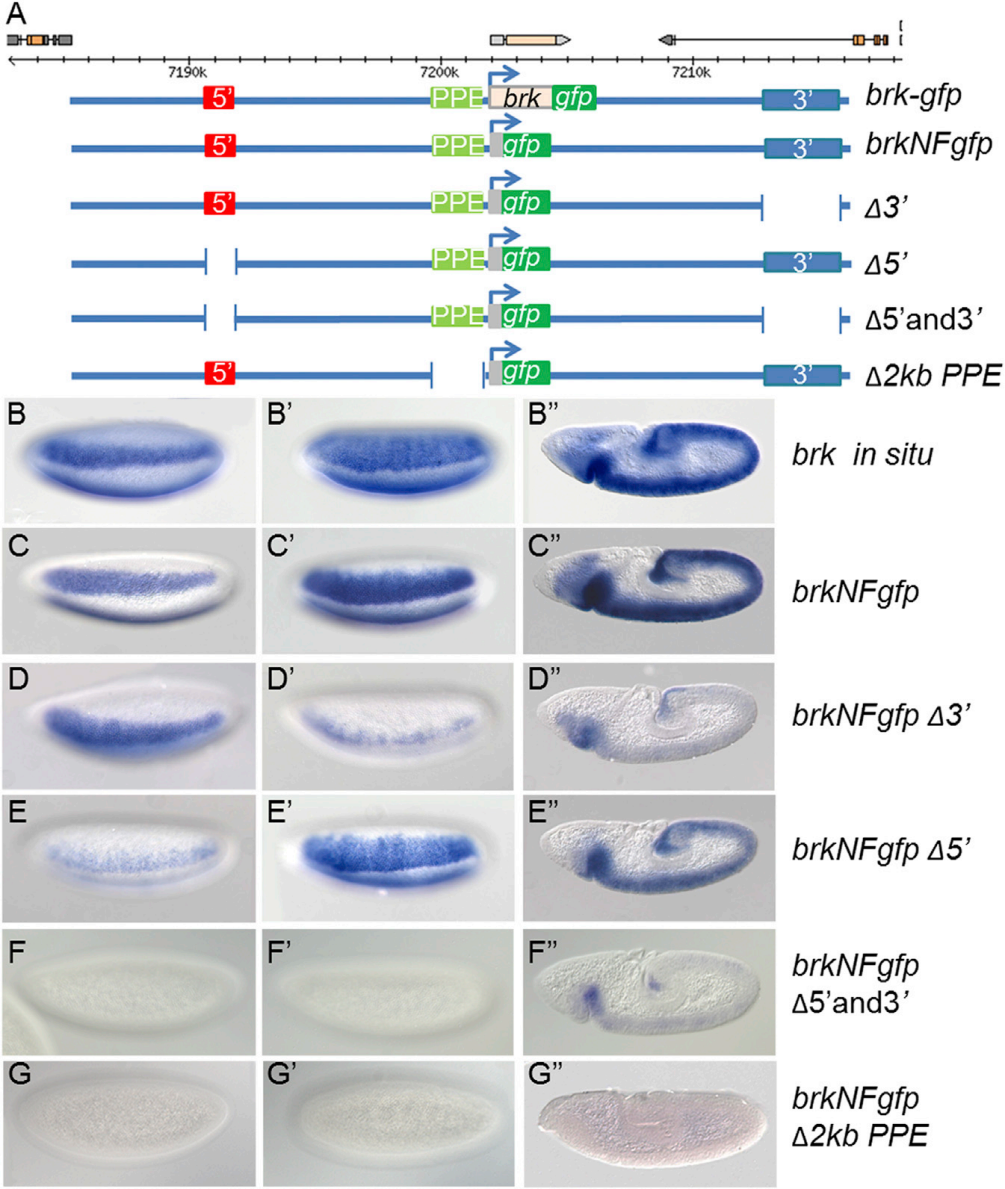
Large Reporter Constructs Show Distinct Roles for Three Early CRMs (A) We created a 32 kb reporter construct, which encompassed the three identified early CRMs and surrounding sequence (extent shown by blue lines in A) and was able to rescue the mutant phenotype. All but the first 66 amino acids of the *brk* coding sequence was replaced by *gfp*, creating a nonfunctional reporter construct used for *cis*-regulatory analysis. Deletions of each of the CRMs were made where indicated by breaks in the blue line. (B–G) In situ hybridization was performed using riboprobes to detect either *brk* transcript in wild-type embryos (B) or *gfp* transcript in transgenic embryos (C–G). The reporter construct expression patterns were compared to the endogenous *brk* pattern at three stages of development: precellularization (B–G), cellularization (B′–G′), and gastrulation (B″–G″). In this and subsequent figures, embryos are oriented with anterior to the left, dorsal up, and are ventrolateral surface views. See also Figure S1.

To examine the function of these CRMs in native context, we constructed a large 32 kb *brk-gfp* rescue construct spanning the *brk* gene and associated flanking sequence and including both distally located early embryonic CRMs (Figure 1A). The *gfp* gene was inserted as an in-frame insertion to the Brk C terminus, thereby creating an ∼32 kb “*brk-gfp*” transgene that supports the viability of *brk* mutants to adulthood (see Experimental Procedures). In a second construct, based on modification of the first, the coding sequence of *brk* was replaced with *gfp* generating a transgene encoding a nonfunctional (NF) *brk* (*brkNFgfp*), which allowed comparison of reporter expression versus that of endogenous *brk* (Figures 1B and 1C). These large reporter constructs facilitate CRM dissection in the context of the genomic locus: the *brk* promoter is retained and enhancer sequences are located in their native positions relative to the promoter and each other.

Recombineering was used to delete the three putative *cis*-regulatory sequences from the *brkNFgfp* large transgene (see Experimental Procedures). When the 3′ CRM sequence was deleted (*brkNFgfp Δ3′*), expression of the reporter was normal early at precellularization (Figure 1D) but lost later at cellularization and gastrulation (Figures 1D′ and 1D″, respectively). In contrast, the opposite trends were observed when the 5′ CRM sequence was deleted (*brkNFgfp Δ5′*); expression was lost at the early time point (Figure 1E) but appeared normal (i.e., matching endogenous *brk*) at later stages (Figures 1E′ and 1E″). When both CRMs were deleted, most embryonic expression during these stages was lost except for weak staining at the anterior in gastrulating embryos (Figures 1F–F″), suggesting that these CRMs are required to support the majority of *brk* expression in the early embryo and that other sequences cannot compensate in their absence.

**Figure 2:**
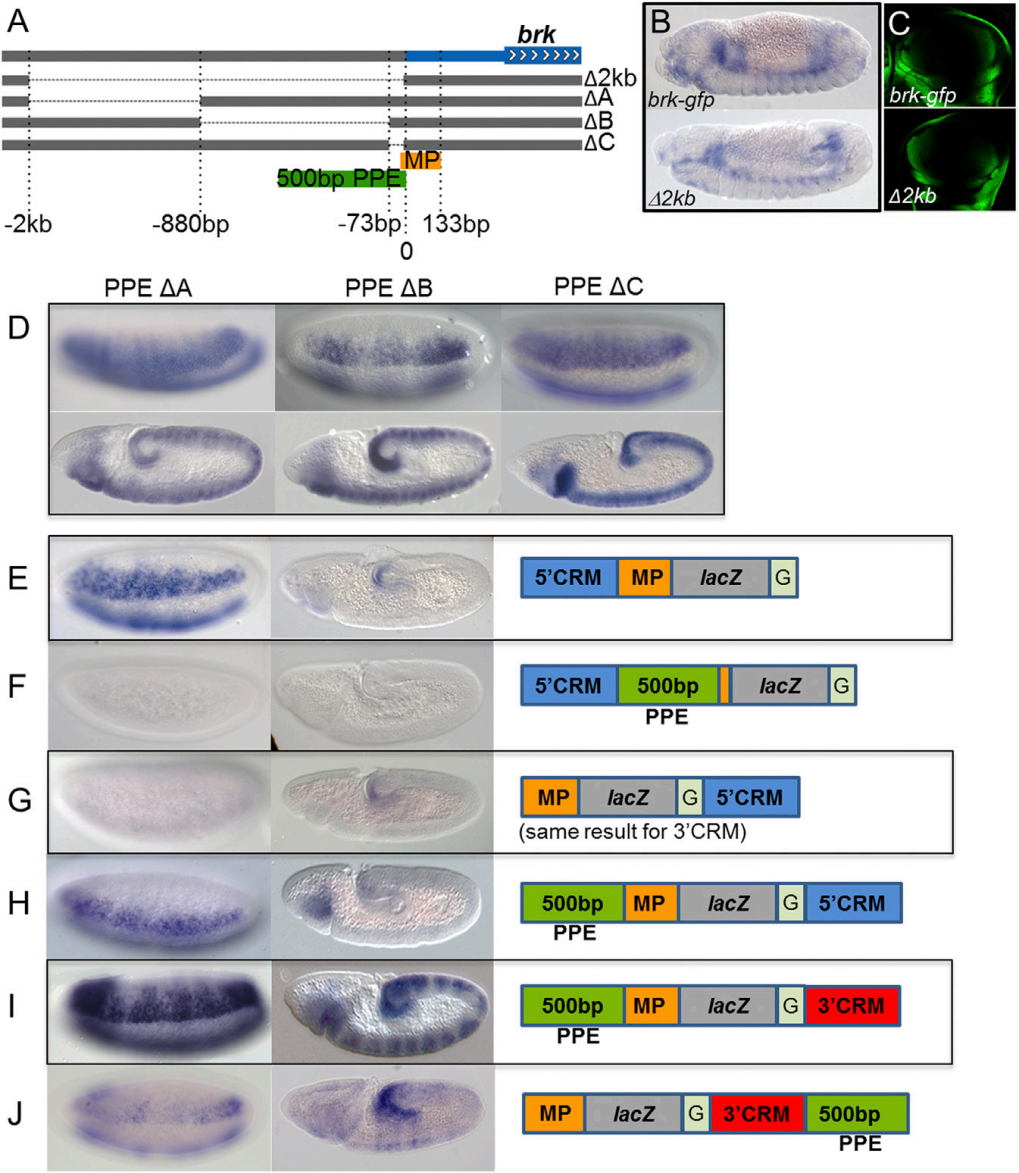
PPE Is Required for Early Embryonic Expression of *brk* and Can Act as an Insulator Bypass Signal (A) The 2 kb PPE deletion was divided into parts (deletions are shown as dotted lines) and these individual deletions were made from the full-length *brkNFgfp* construct. The minimal promoter (MP) used in reporter constructs is from −35 to 133 bp (orange), with 0 representing the transcription start site. (B and C) Later embryonic expression (B) and wing disc expression (C) from *brkNFgfp Δ2kb PPE* and *brkNFgfp* were comparable, as detected by in situ hybridization with a riboprobe to *gfp* for embryonic expression and live imaging of *gfp* to detect expression in wing discs. (D) Patterns of expression from the smaller PPE deletions were visualized by in situ hybridization with a *gfp* riboprobe at cellularization (upper) and gastrulation (lower). (E–J) In situ hybridizations of reporter constructs using a *lacZ* riboprobe are shown at precellularization (E–H) or cellularization (I and J) on the left and gastrulation on the right.

Deletion of the 2 kb fragment encompassing the promoter-proximal region (i.e., PPE) from our large reporter construct (*brkNFgfp Δ2kb PPE*) exhibited a strong phenotype: no expression of the *gfp* reporter was supported at any of these examined stages (Figures 1G–1G″). However, expression of *gfp* within late embryos and in the wing disc, which is driven by different CRMs (Yao et al., 2008), was detected (Figures 2B and 2C). To further investigate the role of the PPE in the early embryo, smaller deletions of this 2 kb segment were made in the context of the *brkNFgfp* large reporter construct and assayed (Figure 2A). In all deletions examined, expression was once again supported, suggesting that some degree of functional redundancy is encoded by this stretch of DNA (Figure 2D). The 2 kb PPE deletion removes a few base pairs (25 bp) of what is defined as the minimal promoter by modENCODE (http://www.modencode.org), but the promoter is not likely affected because the PPEΔC, which removes the most promoter-proximal sequence including these 25 bp, supports expression (Figure 2D). Collectively, these results demonstrate that (1) the 5′ and 3′ CRMs act to support gene expression in a temporal series; (2) the PPE is required to support the activity of 5′ and 3′ CRMs; and (3) the role of the PPE is distinct from that of the minimal promoter.

As the 5′ and 3′ CRMs are located at a distance from the *brk* promoter and the PPE is required to support their function, we hypothesized that the PPE might be required to support long-distance action of the CRMs. To test this idea, we assayed the requirement for the PPE in a standard reporter assay. When the CRMs are placed directly upstream of the minimal promoter, reporter expression is supported even in the absence of the PPE (Figure 2E; data not shown). Placing the CRMs in front of the most promoter-proximal 500 bp of the PPE does not support any expression, indicating that the PPE cannot act as a promoter (Figure 2F). However, when the CRMs are relocated downstream of *lacZ*, which is ∼2 kb in length, the CRMs support little to no activation through the minimal promoter alone (data not shown). Moreover, we found that inserting the Gypsy insulator sequence (Cai and Levine, 1995) in between the *lacZ* and the CRMs further dampens expression, such that none is detectable (Figure 2G; data not shown). However, when the PPE is added just upstream of the minimal promoter, as organized at the endogenous locus, then both CRMs are able to support gene expression despite disadvantaged positioning behind an insulator (Figures 2H and 2I). In contrast, when the PPE is added just downstream of the CRMs, only very weak expression is observed (Figure 2J). Collectively, these results suggest that the PPE supports long-range action of the 5′ and 3′ CRMs and provides “anti-insulator” activity when positioned near the promoter.

**Figure 3:**
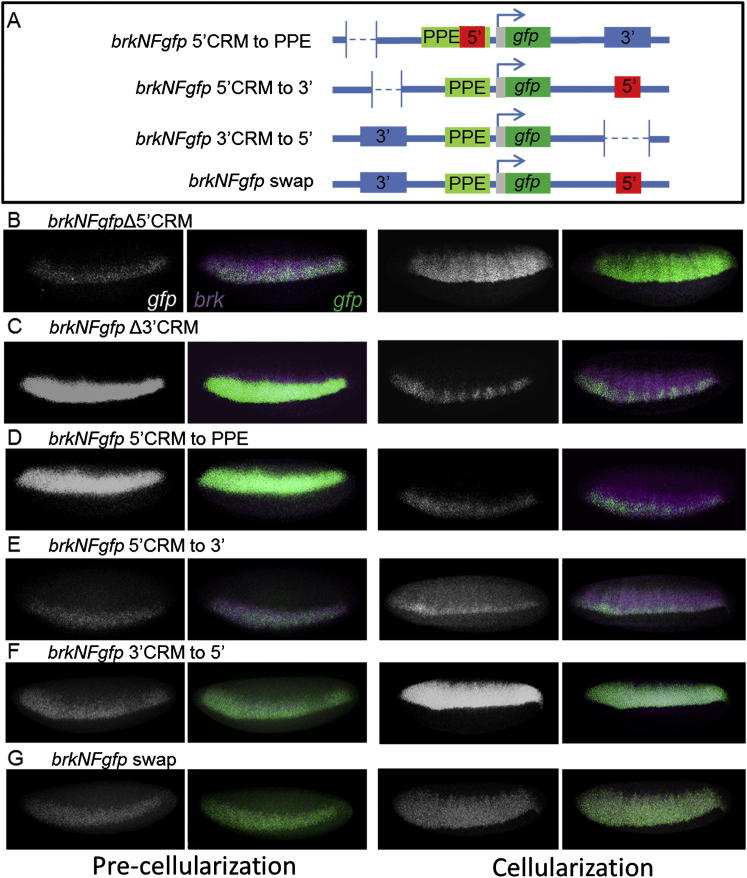
Chromosomal Location of CRMs Affects the Timing of Activation (A) Schematics of the 5′ CRM to PPE and the constructs that translocate the two CRMs are shown. Dotted lines indicate positions of deletions. (B–G) Fluorescence in situ hybridization with riboprobes to *gfp* (white in single-channel images or green in two-color images) and *brk* (purple) was used to compare the expression patterns of these constructs to endogenous *brk* expression. Each construct is shown at two time points, precellularization (left two panels) and cellularization (right two panels). See also Figure S2.

Given the ability of the PPE to support long-range CRM action, we tested the idea that this element might also regulate the exchange from one CRM to the next. The goal was to prolong association of one CRM with the promoter, accomplished by moving the 5′ CRM to the promoter-proximal position using recombineering (i.e., *brkNFgfp 5′ CRM to PPE*; Figure 3A), and to assay how expression was altered relative to endogenous *brk* expression using multiplex in situ hybridization. Through comparison of endogenous *brk* and *gfp* reporter expression, we confirmed that the 5′ CRM is required to support early expression (Figure 3C), whereas the 3′ CRM is required to support late expression (Figure 3B). However, when the 5′ CRM was moved closer to the promoter, placing it in a position where it presumably did not require the PPE for activation (as suggested by our small synthetic constructs; see Figure 2E), the expression of *gfp* associated with the reporter precellularization was normal but at cellularization was deficient relative to that of endogenous *brk* (Figure 3D). Reporter expression from this construct recovers later, at gastrulation, and is able to once again match that of endogenous *brk* (Figure S2C). It is possible that disruption of the PPE by the 1 kb of inserted sequence could lead to the observed loss of 3′ CRM activity at cellularization, although we would argue that this is unlikely, as 3′ CRM expression is seen later in gastrulating embryos (which is dependent on PPE activity; Figure 1G′). We favor the view that by moving the 5′ CRM to the promoter-proximal position, action of the 5′ CRM is prolonged and action of the 3′ CRM is delayed at cellularization.

Reporter expression in precellularized embryos supported by the 3′ CRM alone in a small construct was stronger than expression supported in native context (Figure S1D; compare with Figure 1E). This result suggested to us that the relevant transcription factors are available precellularization to support some expression through the 3′ CRM, but that when located in native context the potential of this sequence to support activation is additionally regulated, possibly by chromatin effects. To further test the idea that chromosomal location of the respective CRMs influences timing of action, we swapped CRM positions and assayed effects on reporter output. We found that moving the 5′ CRM to the location of the 3′ CRM delays activation (Figure 3E, precellularization; compare with Figure 3C), whereas moving the 3′ CRM to the location of the 5′ CRM results in earlier activation (Figure 3F, precellularization; compare with Figure 3B). These results suggest that the earliest activation at the *brk* locus is influenced to some degree by chromosomal location (i.e., a CRM placed in the 5′ position supports earlier expression at precellularization).

However, a complete swap of 5′ and 3′ CRM sequences was also assayed, and this pattern appeared largely normal, narrow in precellularized embryos and broad in cellularized embryos (Figure 3G). If chromatin conformation was driving the pattern independent of CRM sequence identity (i.e., the CRM located at the 5′ position acts first, followed by the CRM located at the 3′ position), the pattern supported by the swap construct would have been expected to be thin at cellularization (i.e., as supported by the 5′ CRM when acting in the 3′ position); but this does not appear to be the case (compare expression at cellularization; Figure 3G; compare with Figure 3C). Therefore, it is likely that in the context of the swap construct when the 3′ CRM gains access to the promoter, which occurs earlier when it is relocated to the 5′ position (e.g., compare expression precellularization; compare Figure 3F with Figure 3B), it remains active through cellularization to support the broad expression observed at this stage and onward at gastrulation. Collectively, these results suggest that although chromosomal positioning does influence timing of CRM action, it is not sufficient to manage which CRM is active and that the CRM sequences themselves contribute.

To interrogate the normal mechanism of switching, namely how the 3′ CRM takes over from the 5′ CRM, we investigated further the idea that interactions of the CRMs and the promoter are regulated temporally using chromatin conformation capture (3C). In a recent study of *brk* locus DNA associations by Chopra et al. (2012), 3C was used to examine interactions of 5′ and 3′ CRMs at the *brk* locus with the promoter at a single time point but in different genetic backgrounds. We investigated whether temporal differences between DNA associations could be discerned using a similarly designed 3C assay conducted at three nonoverlapping time points: (1) 2–2.5 hr (precellularization); (2) 3–3.5 hr (cellularization); and (3) 4–5 hr (gastrulation) (Figures S3A and S3B). Associations between a DNA segment acting as anchor (i.e., the promoter, PPE, and coding sequence) and flanking DNA sequences, including but not limited to 5′ and 3′ CRM segments, were examined. At the early time point, association between the promoter vicinity and 5′ CRM region was indicated, although weak; at the second time point, both the 5′ and 3′ CRMs were found to associate with the promoter area; whereas at the final time point, neither CRM was found to associate with this region (Figure S3B). The 3C experiments suggest that large-scale changes in chromatin conformation do not necessarily accompany the “switch” between 5′ CRM and 3′ CRM action at precellularization to cellularization stages. Whereas both CRMs appear to be in contact with the anchor region at cellularization, local changes in binding may affect the hierarchy of temporal activation between these two CRMs.

**Figure 4:**
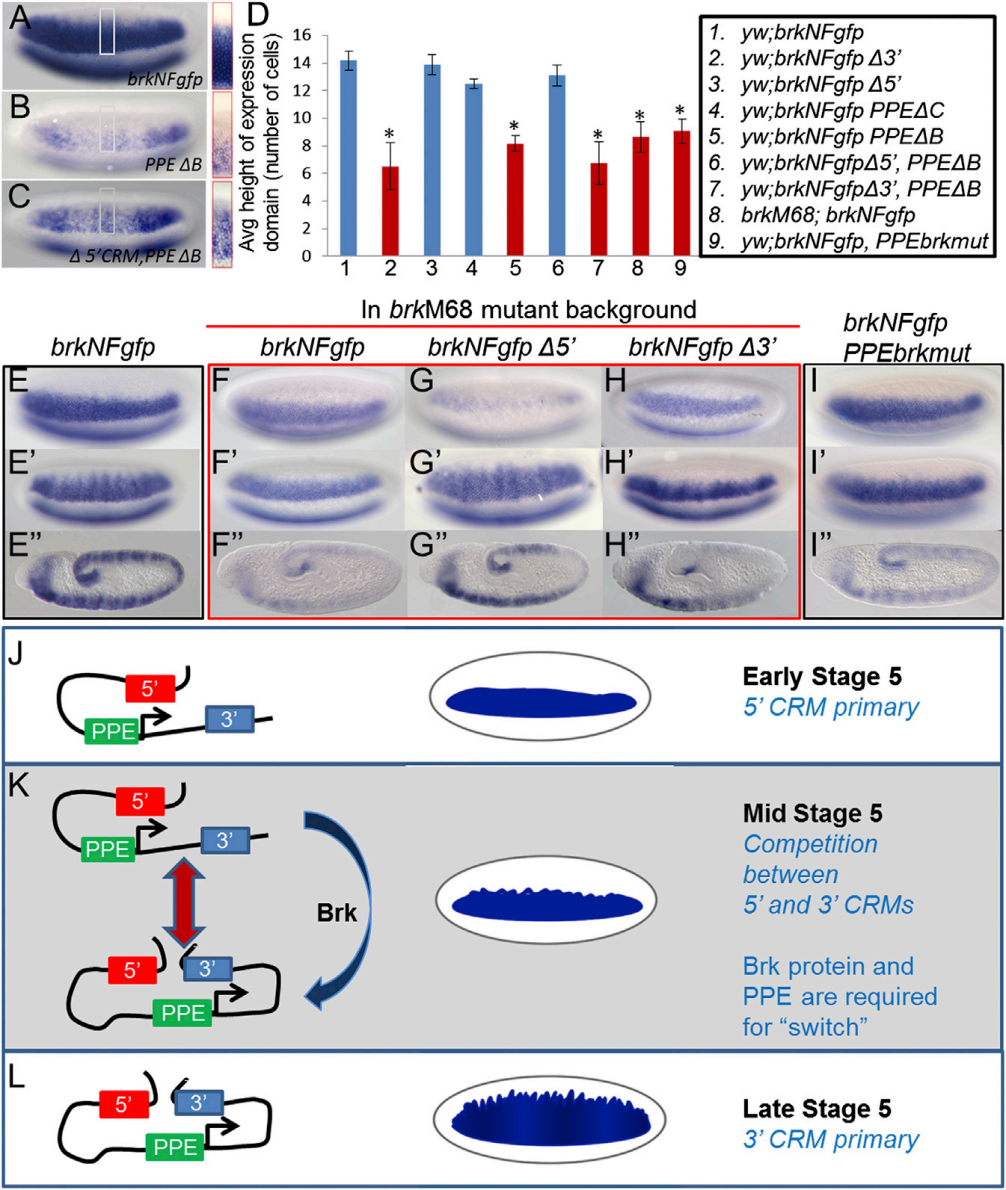
Brk and the PPE Are Required for the Switch from 5′ CRM- to 3′ CRM-Mediated Activation at the *brk* Locus (A–C) Expansion of the expression pattern of the transgenes, as detected by in situ hybridization using a *gfp* riboprobe, at cellularization was measured by counting the number of *gfp*-expressing cells in a specified region at the center of the embryo (gray box; 40× enlargement of the boxed regions is shown to the right). (D) Graph shows the height, in average number of cells, of the *gfp*-expressing domain (see Experimental Procedures), with standard deviations shown with black bars. Those reporters found to be not significantly different from *brkNFgfp* are shown in blue. Those that were significantly different from the full-length reporter (^∗^) but not significantly different from *brkNFgfp* Δ3′ are shown in red (significance was defined at p < 0.001 based on a two-tailed t test). (E–I) Expression of the *gfp* transgenes was assayed in wild-type (E and I) or brk^m68^ mutant backgrounds (F–H), shown at precellularization (E–I), cellularization (E′–I′), and gastrulation (E″–I″). (J–L) Model for regulation of *brk* expression in early embryos. The 5′ CRM is the primary acting module during early stage 5 (precellularization), driving expression in a defined narrow lateral band (J). During cellularization, the 5′ and 3′ CRMs compete for access to the promoter, and Brk protein acts to bias the association toward the 3′ CRM (K); the PPE is also required for a properly timed switch to the later-acting enhancer. By late stage 5, at the completion of cellularization, the 3′ CRM is the primary acting module driving expression of a broad lateral band (L). See also Figure S3.

We then focused attention on the PPE, as this element is required for expression of both distal enhancers, the 5′ and 3′ CRMs, and might provide insight into their temporal action. The PPE likely serves more than one function in the expression of *brk*. We have shown that deleting the whole 2 kb eliminates expression from the reporter (Figure 1G), whereas smaller deletions show varying degrees of reporter expression (Figure 2D), suggesting that the PPE contains partially redundant elements. We used the smaller deletions to identify a region responsible for CRM management. The expression pattern supported by the deletion of the distal 800 bp of the PPE (PPEΔA) or the sequence directly upstream of the minimal promoter (PPEΔC) showed little deviation from wild-type *brk* expression in precellularized (data not shown) or cellularized embryos (Figures 2D and 4D). In contrast, when the 800 bp proximal section of the PPE was deleted (PPEΔB), expression was supported in a narrow stripe that never broadened, even at cellularization (Figure 4D; image in Figure 4B). This expression is very similar to that supported by the *brkNFgfp Δ3′* construct, suggesting that in the absence of this 800 bp sequence the 3′ CRM is impaired. Furthermore, when the 5′ CRM is deleted together with the PPEΔB segment, the pattern broadens to where it is no longer significantly different from full-length expression (Figure 4D; image in Figure 4C). This suggests that in the context of PPEΔB deletion the 3′ CRM was inhibited from acting, but this block is removed upon deletion of the 5′ CRM.

To provide molecular insight into how the switch from one CRM to another is regulated, we dissected transcriptional inputs into the PPE. The modENCODE ChIP data and JASPAR database (Bryne et al., 2008) were used to define a test set of putative DNA-binding factors. Of the genes tested through mutant analysis, expression of *brk* was most affected in the *brk* mutant background itself. Brk has been shown to act as a repressor of transcription (reviewed in Affolter and Basler, 2007). In addition, it was also known that negative autoregulation supports refinement of the *brk* expression domain in the wing disc, but how this is accomplished at a molecular level is not understood (Moser and Campbell, 2005).

When the large reporter constructs were introduced into a *brk* mutant background and assayed, we obtained evidence that Brk protein is required to support the action of the 3′ CRM. When the full-length *brkNFgfp* construct is put into a *brk* mutant background, the expression at cellularization is narrow, similar to that associated with the *brkNFgfp Δ3′* construct (Figure 4F′ compared with Figure 4H′; Figure 4D). In contrast, the *brkNFgfp Δ5′* construct supports normal expansion of the expression domain in the *brk* mutants (Figure 4G′). This indicates that the two CRMs exhibit different relationships to Brk protein levels: the early-acting CRM located upstream of the promoter requires Brk protein be present in order to “shut off,” whereas the late-acting CRM is precluded from acting in *brk* mutants if the early-acting CRM is present. In addition, this phenotype is very similar to that associated with deletion of the PPE proximal segment (i.e., PPEΔB; Figure 4D).

To provide further insight into the mechanism by which Brk supports the CRM switch, ChIP-seq was used to examine Brk occupancy at the *brk* locus at two time points, 2–2.5 hr and 3–3.5 hr, which roughly correspond to when the two CRMs are active (Figures S3C and S3D). Limited occupancy of Brk was detected by Chip-seq at the PPE in 2–2.5 hr embryos, whereas significant Brk occupancy was detected at the PPE in older embryos (3–3.5 hr) (Figure S3D). Brk occupancy was also detected at the 3′ CRM at the later time point, whereas no binding was detected at the 5′ CRM at either of the time points examined (Figure S3C). The Brk 3′ CRM was recently defined as a highly occupied target (HOT) region, bound by many transcription factors (Kvon et al., 2012), whereas the PPE and 5′ CRM are not HOT regions. The majority of transcription factor binding to HOT enhancers is thought to be functionally neutral (Kvon et al., 2012); therefore, we reasoned it more likely that Brk acts through the PPE rather than the 3′ CRM.

Brk binding at the PPE and the loss of 3′ CRM activity in a Brk mutant led us to believe that Brk could be directly acting to mediate switch from 5′ to 3′ CRM activation. It order to directly test this hypothesis, four predicted Brk binding sites located in the vicinity of the PPE proximal half (segment B, two sites, and segment C, two sites; Figure S3E) were mutated in the context of the 32 kb brkNFGFP large reporter (i.e., *brkNFgfp*, *PPEbrkmut*). Expression from this transgene was assayed in a wild-type genetic background, yet the phenotype resembled that of intact wild-type reporter in the *brk* mutant background. Namely, the early pattern, precellularization, was normal, but at cellularization the pattern failed to become broad (Figures 4I and 4I′; compare with Figures 4E and 4E′; see also Figure 4D). These results were confirmed with double in situ hybridization comparing the reporter to endogenous *brk* (data not shown). This result is consistent with the view that Brk acts through the PPE, as mutagenesis of Brk sites in the PPE correlates closely with the *brk* mutant and the PPEΔB phenotypes.

Collectively, our results show that (1) two CRMs control spatially and temporally distinct patterns of *brk* expression; (2) the switch from one CRM to the next requires a promoter-proximal sequence; and (3) levels of Brk protein influence the switch from early enhancer to late-acting enhancer in the early embryo. Although the 5′ CRM is the primary acting module precellularization (Figure 4J), at the onset of cellularization (mid-stage 5) competition between the 5′ and 3′ CRMs for access to the promoter complex is likely (Figure 4K). This competition is affected by Brk protein and the PPE. In the presence of these two factors, the 3′ CRM is able to outcompete the 5′ CRM for access to the promoter (Figure 4L). In the absence of Brk protein, or when the PPE is not intact, the 5′ CRM remains active and blocks the activity of the 3′ CRM. The results of mutagenesis of Brk binding sites within the PPE provide strong evidence for a role for Brk at the PPE; however, the 3C experiments suggest that large-scale changes in chromatin conformation do not necessarily accompany the switch between 5′ CRM and 3′ CRM action. We favor a model in which Brk acts through the PPE to modulate the local 3D chromatin environment to bias 3′ versus 5′ CRM action and thereby catalyze the switch between CRMs.

The general implication of this study is that autoregulatory feedback may afford one CRM a positive advantage in competition with other CRMs for engagement with the promoter. Whether CRM competition is acting to control temporal expression of other genes remains to be determined, but we suggest it is likely. The current view is that important developmental regulators that control large numbers of genes will be autoregulated, because their levels of expression must be tightly controlled (Crews and Pearson, 2009). Autoregulatory control may therefore be a common and effective mechanism used to control temporal gene expression through regulation of sequential activation of CRMs. Once the amounts of a factor rise to a particular level that supports autoregulation, then the timing may be right to switch to a subsequently acting CRM. What better cue to support timing of CRM switch than the factor itself.

## Experimental Procedures

### Fly Stocks

*Drosophila melanogaster* flies of the background *yw* were used as wild-type. The 86Fb attp [*M{vas-int.Dm}ZH-2A,M{3xP3-RFP.attP}ZH*-86Fb] and *Df(1)ED6906, w1118P{3′.RS5+3.3′}ED6906/FM7h* fly stocks were obtained from the Bloomington *Drosophila* Stock Center. *brk^m68^/FM7eve-lacZ* was obtained from Christine Rushlow (New York University) (Jaźwińska et al., 1999b). Viability of the *brk-gfp* construct was tested by introducing this transgene into the heterozygous *brk* mutant background using standard genetic crosses.

### Cloning and Generation of *lacZ* Constructs

Sequences for the 5′ and 3′ CRMs and the PPE were amplified from BAC DNA and cloned into the KpnI site of the eve_*promoter*_-lacZ-attB vector (Liberman and Stathopoulos, 2009). For the insulator bypass assay, the attB vector (Bischof et al., 2007) was modified as stated in the Supplemental Experimental Procedures.

The 86Fb fly stock with attP landing site was injected with reporter constructs in house using standard techniques to generate transgenic lines.

### Generation of 32 kb *brk-gfp* Constructs

The 32 kb *brk* P[acman] construct was generated using recombineering-mediated gap repair as in Venken et al. (2006). The BAC encompassing the *brk* gene (BACR35J16) was obtained from the BacPac Resource Center. Insertion of *gfp* just before the stop codon of *brk* was performed using a *gfp*-sv40-frt-kan-frt plasmid, and the kanamycin (kan) cassette was removed after insertion as in Lee et al. (2001). Deletions and rearrangements of the CRM regions were done using the galK system (Warming et al., 2005). Mutation of the four Brk binding sites was accomplished through a series of fusion PCR reactions using primers PPEmut A–D (Supplemental Experimental Procedures; mutated base pairs are capitalized) and integrated into the large reporter using the galK system. Large reporter constructs were grown and isolated as in Dunipace et al. (2011) and injected into 86Fb flies.

All primers used for gap repair and recombineering are listed in the Supplemental Experimental Procedures.

### In Situ Hybridization

Embryos were fixed and stained following standard protocols. Antisense RNA probes labeled with digoxigenin or FITC-UTP were used to detect reporter or in vivo gene expression as described previously (Jiang and Levine, 1993; Kosman et al., 2004).

### Quantification of Reporter Expression Width

Lateral images of alkaline phosphatase-stained embryos were taken using a 40× objective on an Axioplan microscope. Five to seven embryos of each genotype were then analyzed for expression patterns. A box of 20 μm width was drawn in the center of the anterior-posterior axis, from the ventral border of the *brk* expression domain to the visible dorsal edge of the embryo. All cells expressing the reporter that were partially or completely within this box were counted. This total number was then divided by the width of the box, in number of cells, giving an average height of expression domain. Significance was tested using a Student’s two-tailed t test to compare all reporter domains to that of the full reporter construct (*brkNFgfp*), and separately to compare all constructs to brkNFgfp Δ3′. Significance was designated by a p value of <0.001.

## Supplemental Information

Document S1. Supplemental Experimental Procedures and Figures S1–S3
