# Supplementary material for: Autoregulatory Feedback Controls Sequential Action of cis-Regulatory Modules at the brinker Locus
Source: Dev Cell. 2013 Sep 16;26(5):536–43. doi: 10.1016/j.devcel.2013.08.010 (PMC3782659; doi:10.1016/j.devcel.2013.08.010)
Supplement: Document S1. Supplemental Experimental Procedures and Figures S1–S3 [file mmc1.pdf]

Developmental Cell, Volume *26*

Supplemental Information

Autoregulatory Feedback Controls Sequential Action of *cis*-Regulatory Modules at the *brinker* Locus

Leslie Dunipace, Abbie Saunders, Hilary L. Ashe, and Angelike Stathopoulos

INVENTORY OF SUPPLEMENTAL MATERIALS

Supplemental Figure 1, related to Figure 1

Supplemental Figure 2, related to Figure 3

Supplemental Figure 3, related to Figure 4

Supplemental Experimental Procedures

Supplemental References

Supplemental Figure 1, related to Figure 1: Two distal CRMs surrounding *brk* drive expression in distinct spatio-temporal patterns. **(A)** Twist ChIP-seq defined binding [shown in reads per million, RPM, published previously: (Ozdemir et al., 2011)] was identified previously in 3 domains: 10kb upstream (5' CRM), promoter proximal (PPE), and 8.5KB downstream (3' CRM). **(B-E)** We made reporter constructs of each of the defined regions by placing them upstream of an *eve* minimal promoter driving *lacZ*. In situ hybridization was performed using riboprobes to detect *lacZ* transcript in transgenic embryos (C-E). The reporter construct expression patterns were compared to the endogenous *brk* pattern at three stages of development: precellularization (B-E), cellularization (B'-E') and gastrulation (B''-E'').

Figure S1

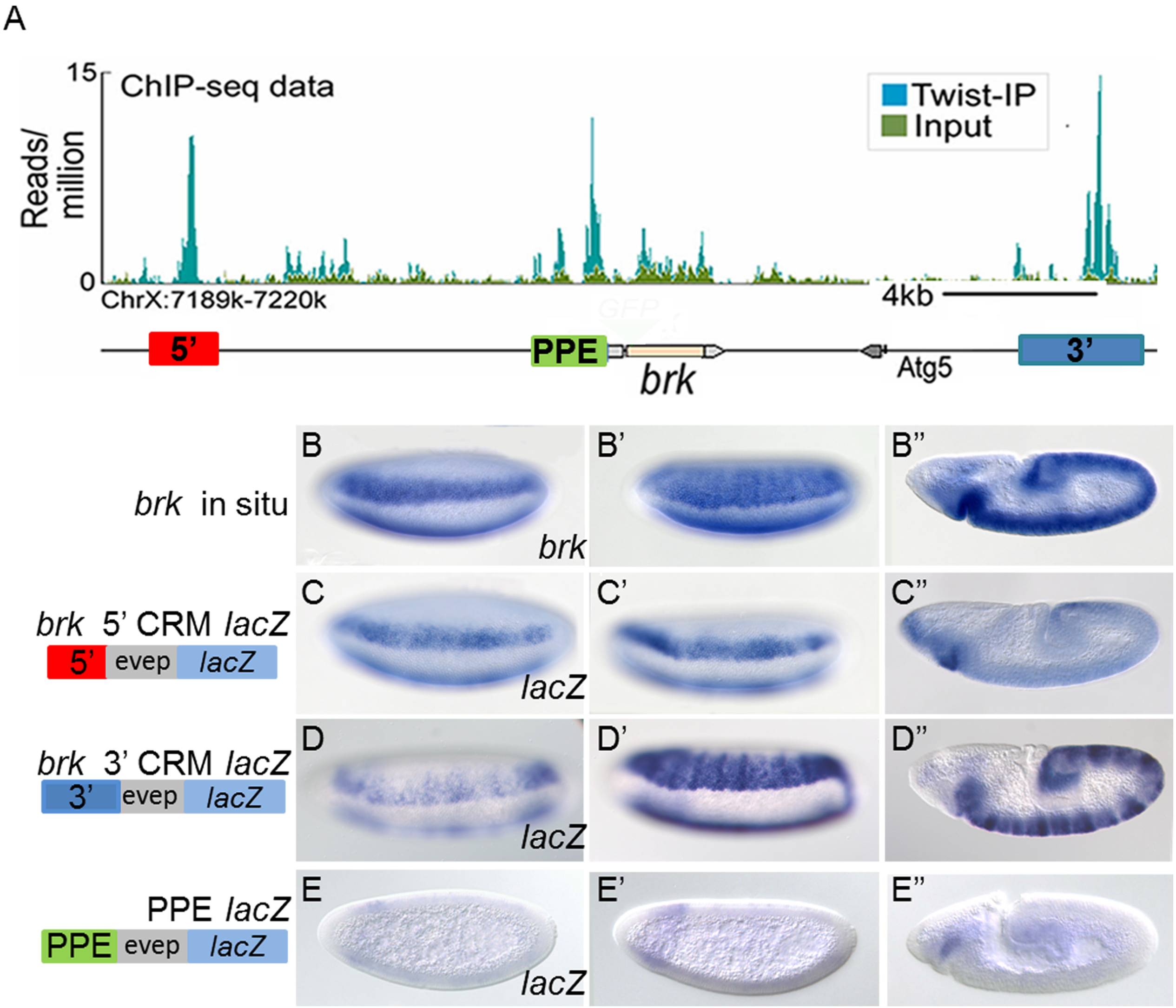
A
15
ChIP-seq data
Twist-IP
Input
Reads/ million
0
ChrX:7189k-7220k
4kb
5'
PPE
brk
Atg5
3'
brk in situ
B
B'
B"
brk
brk 5' CRM lacZ
5' evep lacZ
C
C'
C"
lacZ
brk 3' CRM lacZ
3' evep lacZ
D
D'
D"
lacZ
PPE lacZ
PPE evep lacZ
E
E'
E"
lacZ

Figure S2, related to Figure 3: Chromosomal location of CRMs also affects the timing of activation in embryos at gastrulation. **(A-F)** Two color in situ hybridization using *gfp* and *brk* riboprobes. The pattern of expression of *gfp* (white, in one color images on the left, or green, in two color images on the right) is compared to endogenous *brk* expression (purple). In the *brkNFgfp*Δ5'CRM the reporter and endogenous expression patterns are completely overlapping (A), while the *brkNFgfp*Δ3'CRM shows a much reduced expression pattern compared to endogenous (B). Although the late stage 5 expression of *brkNFgfp*5'CRM to PPE was deficient compared to *brk*, by stage 9 the expression pattern again mimics the endogenous (C). Moving the 5' CRM to the 3' position allows for later expression of the 5' CRM than exhibited from its endogenous position (D). Moving the 3' CRM to the 5' position, however, does not affect its ability to act at this later stage (E). The *brkNFgfp* swap construct shows complete overlap of *gfp* and *brk* patterns at gastrulation (F).

Figure S2

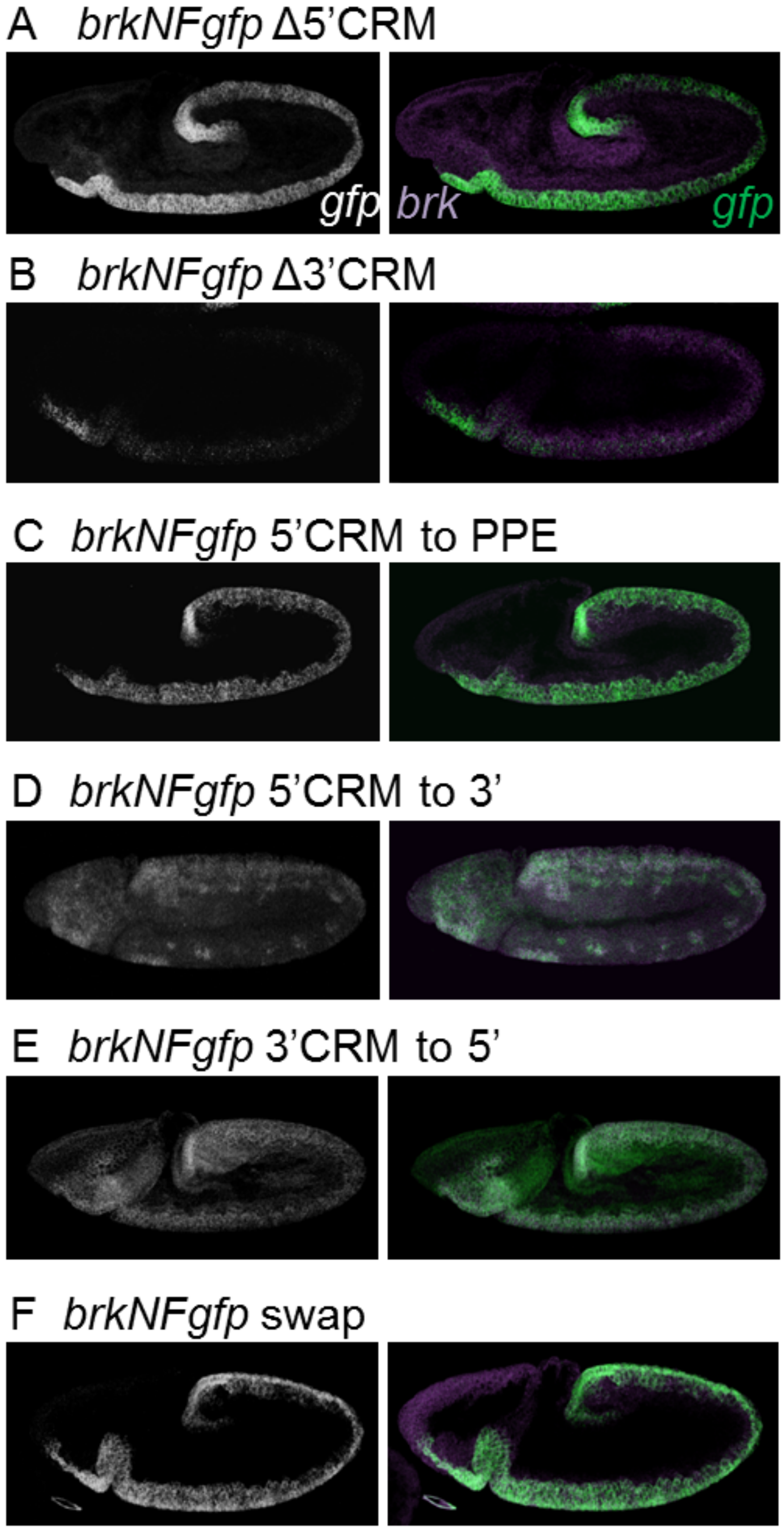
A brkNFgfp Δ5'CRM
gfp
brk
gfp
B brkNFgfp Δ3'CRM
C brkNFgfp 5'CRM to PPE
D brkNFgfp 5'CRM to 3'
E brkNFgfp 3'CRM to 5'
F brkNFgfp swap

Figure S3, related to Figure 4: Chromosome conformation capture (3C) and Brk ChIP-seq data at the *brk* locus.

(A) Twelve primers sets (indicated by grey arrows, primer names written below) were designed spanning a 44kb locus in which one primer was just downstream of an EcoRI restriction site and the other primer in each pair was just downstream of the restriction site defining the "Anchor Point". The anchor point encompasses the PPE as well as the promoter and the gene itself. The black tick marks indicate the site of EcoRI digestion and the numbers given are the distance from the transcription start site (Kb). **(B)** 3C was performed using cross-linked embryos from 2-2.5 hour (blue line), 3-3.5 hour (red line) and 4-5 hour (green line) aged collections. The locations of the 5' and 3' CRMs are highlighted in grey and the anchor point is highlighted in blue. Measurements of the normalized average intensity are mapped to the location of the nearest EcoRI restriction site and represent the degree of interaction between the downstream fragment and the anchor point. Standard deviations for each of the points are given by the black bars.

(C) Brk (Brk) and corresponding pre-immune (PI) ChIP-seq tracks are shown for 2-2.5 hr and 3-3.5 hr embryos for the entire *brk* locus. **(D)** The lower image is a zoomed-in version of the area within the dotted blue box, specifically showing the region defined as the PPE. **(E)** The predicted Brk binding sites (red rectangles) are mapped onto the PPE. The sites mutated in the brkNFgfpPPEmut construct are marked with an asterix.

Figure S3:

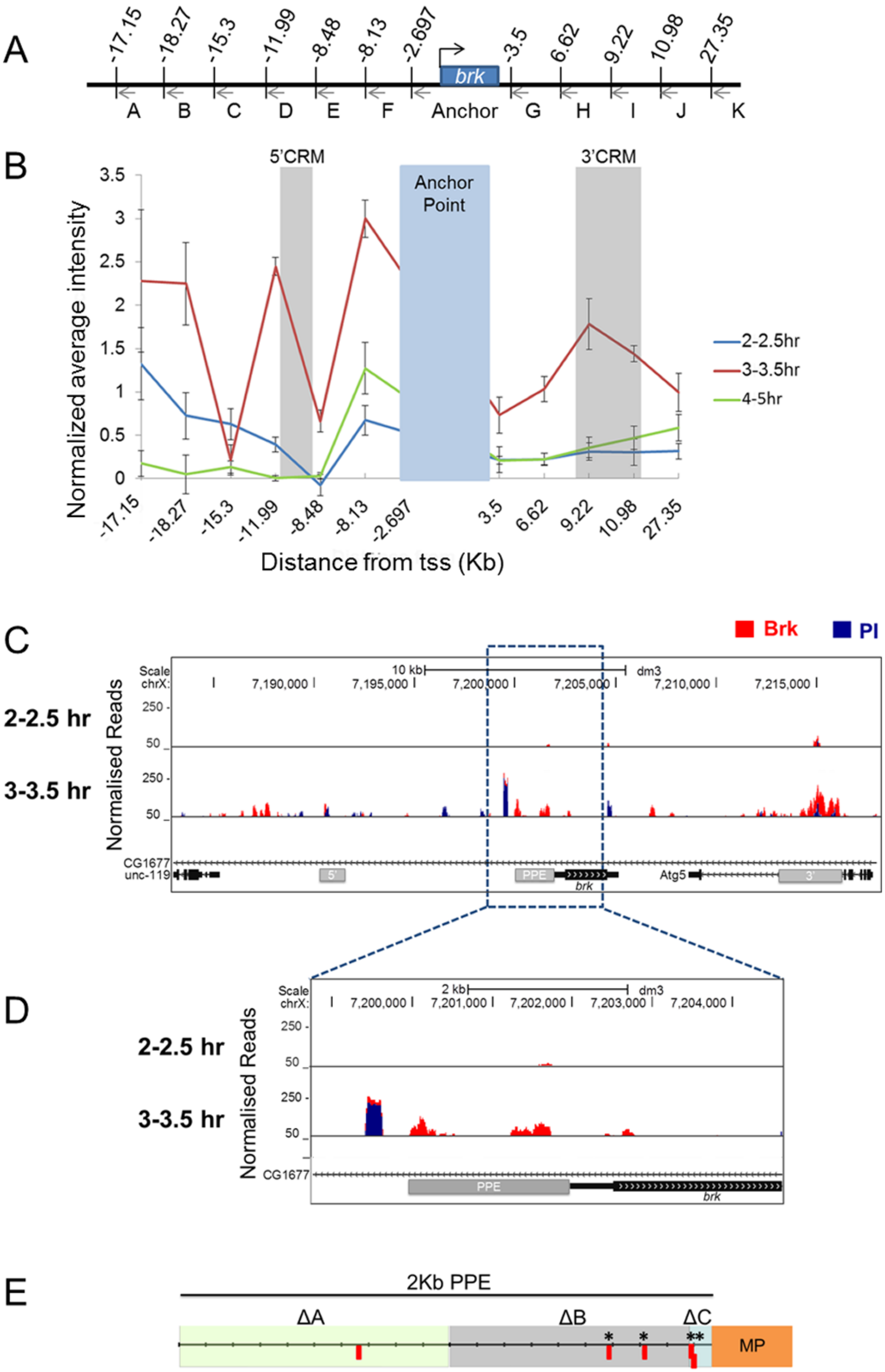
A
-17.15
-18.27
-15.3
-11.99
-8.48
-8.13
-2.697
brk
-3.5
6.62
9.22
10.98
27.35
A B C D E F Anchor G H I J K
B
5'CRM
Anchor Point
3'CRM
Normalized average intensity
Distance from tss (Kb)
2-2.5hr
3-3.5hr
4-5hr
C
Brk
PI
2-2.5 hr
3-3.5 hr
Normalised Reads
Scale
chrX:
10 kb
dm3
7,190,000
7,195,000
7,200,000
7,205,000
7,210,000
7,215,000
CG1677
unc-119
5'
PPE
brk
Atg5
3'
D
2-2.5 hr
3-3.5 hr
Normalised Reads
2 kb
7,200,000
7,201,000
7,202,000
7,203,000
7,204,000
E
2Kb PPE
ΔA
ΔB
ΔC
MP

Supplemental Experimental Procedures:

Cloning and generation of lacZ constructs: To create the vector used in the insulator by-pass assay, the BamHI site in the attB vector (Bischof et al., 2007) was killed and then even-skipped (eve) promoter was amplified from $eve_{promoter}$-lacZ-attB and inserted into the attB plasmid at the NotI site, introducing a StuI site and a BamHI site downstream from the eve promoter. lacZ with an SV40 termination sequence was subcloned from the $eve_{promoter}$-lacZ-attB into the introduced BamHI site in the new plasmid. The gypsy insulator element (Cai and Levine, 1995) was inserted into the plasmid using KpnI/XbaI, and introducing an AvrII and AgeI site downstream. The *brk* minimal promoter and the PPE Tether 500 with *brk* minimal promoter were amplified from BAC DNA and replaced the eve promoter using EcoRI or BglII/StuI. The 5' CRM was amplified from BAC DNA and inserted into the upstream position in the BglII site by cutting with BamHI. The CRMs were inserted in the downstream position using the introduced AvrII/AgeI sites.

Chromatin immunoprecipitation coupled with high-throughput sequencing (ChIP-seq): The Brk antibody was raised in guinea pig using standard procedures at Eurogentec, against MBP-$Brk_{aa251-650}$. Embryo ChIP was performed as described (Sandmann et al., 2006), except that 5% formaldehyde was used in the crosslinking solution. Samples were sonicated for 30 min using a Bioruptor with 9 sec on, 1 min off cycles, on high-energy settings. Sequencing was performed on the ABI SOLiD by the University of Manchester Core Genomic Technologies facility. Sequence reads for duplicate samples were mapped by the University of Manchester Core Bioinformatics facility. The reads shown in Figure S3 were normalized using a correction factor calculated as

the mean of all median values divided by the per sample median value. The full ChIP-seq data is being published elsewhere

Chromatin conformation capture: Timed collections of YW embryos were made by allowing the flies to lay for 0.5 hr (for 2-2.5 and 3-3.5 hour collections) or 1 hr (for 4-5hr collection) and then aging them the appropriate amount of time in a 25˚C incubator. The 3C assay was performed as in Chopra et al. (2012) using 400U of EcoRI per sample. A positive control sample was created using the BAC containing the *brk* locus (BACR35J16), digested with EcoRI and ligated, giving approximately equal molar ratio of all possible ligation combinations. Two biological replicates were made for each time point. Primers were designed 100-150bp downstream from each EcoRI restriction site within the 44kb region, and oriented towards the restriction site. For each primer pair three technical replicates of the semi-quantitative PCR were performed. The PCR products were run on 1.8% LMP agarose gels and background corrected average intensities of each band was determined using the AlphaImager program (Alpha Innotech). Each intensity reading was normalized to the positive control and non-ligated control samples.

Supplemental Experimental Procedures: Primer List

Primer Name	Primer Sequence
brk 5' KpnI-f	aatggtacctcggaaaatacctgcgcatcct
brk 5' KpnI-r	aatggtaccttcacacagccatcgatcgt
brk 3' KpnI-f	tatggtaccgtgccggcgaaggtgtaaatacaa
brk 3' KpnI-r	tatggtaccatcggaacggatcgcaggtcttt
PPE KpnI-f	ttaaggtaccgagcaaaaggaagagagagagagc
PPE KpnI-r	ttaaggtaccgcaaggaatccaaaacacaattgttc
eve-p Not-f	atagcggccgcGAGCGCAGCGGTATAAAAGGG
evep not-r	atagcggccgcggatccaaggcctTCTAGGTCCACGGGACTG
gypsy-KPNI-f	ataggtaccgtatggcaagaaaaggtatgc
gypsy-xbaI-r	atttctagacctaggaccggtcaatttattcgcaaaaacattg
PPE500bp-BGLII-F	aacAGATCTgggtgactgtggcaatcgaaaaga
PPE500bp-STUI-R	aacAGGCCTaattgttctgccctcggcgttcta
brk5 ageI-f	aatacggttcggaaaatacctgcgcatcct
brk5 avrII-r	aatcctaggttcacacagccatcgatcgt
MP stuI-r	aataggcctTATATGTCTGCGTGCTGTTGC
MP RI-f	aatgaattcgaacgtatcttcgaactagaacg
Brk3'sm-ageI	tatACCGGTTCACCATGCTCTTTGCCTCC
Brk-3'sm-avrII	tatcctaggCGCAGCACCTGCAACATTAG

Primer Name	Primer Sequence
brk5 bamHI-f	aatggatcctcggaaaatacctgcgcatcct
brk5 bamHI-r	aatggatccttcacacagccatcgatcgt
brk-la-f	aggcgcgcctacgaccaacgttgccacatactc
brk-la-r	cgcggatcccagcatttctcgcagtgcacaa
brk-ra-f	cgcggatccagtgcctgcggtaattgatagc
brk-ra-r	cggttaattaaacctcgactagggtacacaaactg
brk gfp f	attctcgtggcggatactgcgcccacaaacctgaccctggtggccggtggaggcggtgggggtatgagcaagggcgaggaact
brk-gfp-r	gactgccattataacatccgattggtgggattgctgcttccttagttcgaagagctattccagaagtagtga
brk-galk3'-f	tatatatgcagcaggcgggggagaagaggagagtcacggagtaggagttgcctgttgacaattaatcatcggca
Brk-galk3'-r2	acaaatacaaatacaaataggaatagaagatataataggagattctactaTCAGCACTGTCCTGCTCCTT
brk-del3'-c	TATACGTCGTCCGCCCCCTCTTCTCCTCTCAGTGCCTCATCCTCAACCTCATCATCTTAGAGGATAATATAGAAGATAAGGATAAACATAAACATAAACA
brk-del3'-f	atatgcagcaggcgggggagaagaggagagtcacggagtaggagttggagtagtagaatctcctattatatcttctattcctatttgtatttgtatttgt
brk-galk5'-f	tccaaaagacccgatgaaggatcgaacaggtactacgatgatattggtcgcctgttgacaattaatcatcggca
brk-galk5'-r	aacttgttttcacacagccatcgatcgtcgattgtcgatcaaactgatgttcagcactgtcctgctcctt
brk-del5'-c	AGGTTTTCTGGGCTACTTCCTAGCTTGTCCATGATGCTACTATAACCAGCTGTAGTCAAACTAGCTGTTAGCTGCTAGCTACCGACACACTTTTGTTCAA

Primer Name	Primer Sequence
brk-del5'-f	tccaaaagacccgatgaaggatcgaacaggtactacgatgatattggtcgacatcagtttgatcgacaatcgacgatcgatggctgtgtgaaaacaagtt
brkNF-del-c	GGGTGAAATTCGACGTTCAGGAGCTTAGTATGTCCTTACTATTACTAACGCCACCTCCGCCACCCCCATACTCGTTCCCGCTCCTTGACAAGTGACCGCA
brkNF-del-f	cccactttaagctgcaagtcctcgaatcatacaggaatgataatgattgcggtggaggcggtgggggtATGAGCAAGGGCGAGGAACTGTTCACTGGCGT
brkNF-galk-f	cccactttaagctgcaagtcctcgaatcatacaggaatgataatgattgccctgttgacaattaatcatcggca
brkNF-galk-r	ACGCCAGTGAACAGTTCCTCGCCCTTGCTCATacccccaccgcctccacctcagcactgtcctgctcctt
brk-PPE2kb-galk-r	gcgtttgcgatccgcacggcaaggaatccaaaacacaattgttctgcccttcagcactgtcctgctcctt
brk-PPE2kbgalk-f	cagcagagacgtgggaatctgaaccccagatatctatgtatagttgttgccctgttgacaattaatcatcggca
brk-del2kbPPE-f	cagcagagacgtgggaatctgaaccccagatatctatgtatagttgttgcagggcagaacaattgtgttttggattccttgccgtgcggatcgcaaacgc
brk-del2kbPPE-c	gtcgtctctgcacccttagacttggggtctatagatacatatcaacaacgtcccgtcttgttaacacaaaacctaaggaacggcacgcctagcgtttgcg
PPEdelB-galk-f	ggtattatggcatcccgctcgcacttgcactccaagcacgcacacatgcacctgttgacaattaatcatcggca
PPEdelB-galk-r	tgagcattgcagacaaactgacgacaatcgtgtcgcgaggtgtctgcgcttcagcactgtcctgctcctt
5'CRMtoPPE-galk-f	ggtattatggcatcccgctcgcacttgcactccaagcacgcacacatgcacctgttgacaattaatcatcggca

Primer Name	Primer Sequence
5'CRMtoPPE-galk-r	tgagcattgcagacaaactgacgacaatcgtgtcgcgaggtgtctgcgcttcagcactgtcctgctcctt
5'CRMtoPPE-f	ggaatgggttgctccgtcccatgttgtccattcggtcccaccgtgcacactactacgatgatattggtcg
5'CRMtoPPE-r	tcacgcacacaggcgtaaagtgcctgcctgtctgctgtgcgctgctgttgttgtcgatcaaactgatgt
PPEdelB-f	gctgccagcaaagggttaagtgtgggcctactaatacatgtaaaaaactagtcgacggcggcgccggcagcgacgc cggccgaggcgaggcagtctagaa
PPEdelB-c	cgacggtcgtttcccaattcacacccggatgattatgtacatttttgatcagctgccgccgcggccgtcgctgcggcc ggctccgctccgtcagatctt
PPEdelA-galk-f	cagcagagacgtgggaatctgaaccccagatatctatgtatagttgttgccctgttgacaattaatcatcggca
PPEdelA-galk-r	tagtttttacatgtattagtaggcccacacttaaccctttgctggcagctcagcactgtcctgctcctt
PPEdelA-f	cagcagagacgtgggaatctgaaccccagatatctatgtatagttgttgcgctgccagcaaagggttaagtgtgggcc tactaatacatgtaaaaaacta
PPEdelA-c	gtcgtctctgcacccttagacttggggtctatagatacatatcaacaacgcgacggtcgtttcccaattcacacccggat gattatgtacatttttgat
PPEdelC-galk-f	caggcaggcactttacgcctgtgtgcgtgagcatttcagagtgaaccgcacctgttgacaattaatcatcggca
PPEdelC-galk-r	gcgtttgcgatccgcacggcaaggaatccaaaacacaattgttctgcccttcagcactgtcctgctcctt
PPEdelC-f	caggcaggcactttacgcctgtgtgcgtgagcatttcagagtgaaccgcaagggcagaacaattgtgttttggattcctt gccgtgcggatcgcaaacgc
PPEdelC-c	gtccgtccgtgaaatgcggacacacgcactcgtaaagtctcacttggcgttcccgtcttgttaacacaaaacctaagga acggcacgcctagcgtttgcg
PPEmutA-f	gctgccagcaaagggttaagt
PPEmutA-r	AGttttgttgctgctgtgccgtgctgtgctgagtt

Primer Name	Primer Sequence
PPEmutB-r	gcattgcagacaaactgacgaca
PPEmutB-f	gcacagcagcaacaaaaCTATAGcaaaagggaactggtattatgg
PPEmutC-r	tcggcTATCTAGTctgcATAGATATCcgtcgactgcggttcactc
PPEmutC-f	tgtctgcaatgctcagcttcaCTATAGtgcgctctctctctgttgctcg
PPEmutD-f	CTAGATAgccgaggcgaggcagtctagaa
PPEmutD-r	TATATGTCTGCGTGCTGTTGC
3C-Anchor	AAAAGGCGAGAGAAATACATTTTG
3C-A	GCTATCAGTTCACACCGGCATTTC
3C-B	TTCATTTGTTGCTGTCGTCGTCGGTTC
3C-C	TGAAACCGAATCCTGCCTATGG
3C-D	cgaggagcccatgtcactatggtgtacaat
3C-E	ACACCATTCGTTACCACAACTC
3C-F	AGAAAGGGAGTGAGCAAGCGAGAG
3C-G	CGCGAGATTCGTTCCTGTGATTAC
3C-H	AGCCCTGGCGTTAAATTGATTGG
3C-I	tgcaagtggctttcagtgttgccaagtt
3C-J	GAACCAGAGCGGGACAGAGAGATCAGAG
3C-K	TGGGAGTAAACACAACCGTTCG

Supplemental References:

Sandmann, T., Jakobsen, J.S., and Furlong, E.E. (2006). ChIP-on-chip protocol for genome-wide analysis of transcription factor binding in Drosophila melanogaster embryos. Nat Protoc *1*, 2839-2855.
